# Liquid Biopsy Serial Monitoring of Treatment Responses and Relapse in Advanced Esophageal Squamous Cell Carcinoma

**DOI:** 10.3390/cancers12061352

**Published:** 2020-05-26

**Authors:** Josephine Mun Yee Ko, Hoi Yan Ng, Ka On Lam, Keith Wan Hang Chiu, Dora Lai Wan Kwong, Anthony Wing Ip Lo, Jean Chrysei Wong, Roger Chia Wei Lin, Henry Chun Hung Fong, Jason Ying Ki Li, Wei Dai, Simon Law, Maria Li Lung

**Affiliations:** 1 Department of Clinical Oncology, University of Hong Kong, Hong Kong, China; joko@hku.hk (J.M.Y.K.); biancang@connect.hku.hk (H.Y.N.); lamkaon@hku.hk (K.O.L.); dlwkwong@hku.hk (D.L.W.K.); ever5js@hku.hk (J.C.W.); rogerlin@hku.hk (R.C.W.L.); fchhenry@hku.hk (H.C.H.F.); ykjli@hku.hk (J.Y.K.L.); weidai2@hku.hk (W.D.); 2 Department of Diagnostic Radiology, University of Hong Kong, Hong Kong, China; kwhchiu@hku.hk; 3 Division of Anatomical Pathology, Queen Mary Hospital, Hong Kong, China; awilo.qmh@gmail.com; 4 Department of Surgery, University of Hong Kong, Hong Kong, China; slaw@hku.hk

**Keywords:** circulating tumor cells, cell-free DNA, CTC enumeration, longitudinal real-time monitoring, non-invasive biomarker, liquid biopsy, advanced ESCC, early prediction, prognosis, chemotherapy

## Abstract

(1) Background: Early predictive markers to track treatment responses are needed for advanced esophageal squamous cell carcinoma (ESCC) patients. We examined the prognostication and risk stratification role of liquid biopsy serial monitoring for this deadly cancer. (2) Methods: Circulating tumor cells (CTCs) and plasma cell-free DNA (cfDNA) were isolated from 60 ESCC patients treated by chemotherapy (CT) at five serial timepoints: baseline (CTC1/cfDNA1), CT pre-cycle III (CTC2/cfDNA2), CT post-cycle IV, end of CT and relapse. (3) Results: In 45/57 ESCC patients with evaluable CTC counts at CT pre-cycle III, positive CTC2 (≥3 CTCs) is independently associated with response at interim reassessment and progression-free survival (PFS) in multivariate analysis. In 42/57 ESCC patients with changes of CTC1/CTC2 and cfDNA1/cfDNA2, patients categorized into four risk groups based on the number of favorable and unfavorable changes of CTC1/CTC2 and cfDNA1/cfDNA2, were independently associated with overall survival (OS) by multivariate analysis. (4) Conclusions: CTC counts at pre-cycle III are independently associated with response at interim reassessment and PFS. Combined changes of CTC counts and cfDNA levels from baseline to pre-cycle III are independently associated with OS. Longitudinal liquid biopsy serial monitoring provides complementary information for prediction and prognosis for CT responses in advanced ESCC.

## 1. Introduction

Esophageal cancer (EC) is a major cause of cancer morbidity and mortality among Chinese, and is one of the most aggressive malignancies with a dismal five-year survival rate for both, locoregional and metastatic disease [1,2]. The two histological subtypes, including esophageal squamous cell carcinoma (ESCC) and adenocarcinoma, show different characteristics regarding epidemiology, pathogenesis, tumor biology and therapeutic strategies. The majority of EC cases occurs in Asia [3]. In contrast to Western countries, the predominant histological subtype in Chinese is ESCC. ESCC patients present at advanced stage with poor outcomes, since early disease is often asymptomatic. Despite improved survival for locoregionally advanced ESCC patients receiving chemoradiotherapy (CRT) alone or prior to curative resection, disease relapse and drug resistance are common [4,5,6,7]. For advanced metastatic ESCC, the application of clinical stage groups assessed by conventional imaging and clinical examination for prognosis is insufficient. To improve treatment outcomes, there is an unmet need for the development of biomarkers that allow timely detection of treatment resistance and selection of subsequent therapy. We aim to investigate the clinical utility of liquid biopsies in regionally advanced and metastatic ESCC.

Circulating tumor cells (CTCs), released into the blood circulation from the primary tumors or metastatic lesions, are considered as the metastatic seeds [8,9]. Increasing evidence indicates CTCs are prognostic indicators for metastatic relapse in various cancers [10,11,12,13,14]. Among the numerous CTC enrichment methods developed, the epithelial cell adhesion molecule (EpCAM)-based enrichment is the most commonly used [9,15]. However, accumulating evidence shows that CTCs are highly heterogeneous. The use of antibody-based capture for CTC enrichment from CellSearch^®^ (Menarini Silicon Biosystems, Pennsylvania) and others may underestimate CTC numbers. This will negatively impact the significance of CTC enumeration, as this method cannot detect the CTC sub-populations that have low EpCAM expression and non-epithelial phenotypes [16]. This latter subgroup is particularly interesting as this group of CTCs includes epithelial-to-mesenchymal transition (EMT) cells, hybrid CTCs with both epithelial/EMT+, irreversible EMT+ tumor cells, and circulating tumor stem cells (CTSCs) [17,18]. There is significant criticism about its relatively low sensitivity and specificity. An earlier ESCC CTC analysis employing the CellSearch system reported baseline CTCs were detected at 27.8% [19]. Therefore, we chose the sized-based CTC enrichment strategy because it can isolate viable CTCs, with superior sensitivity, and is independent of cell surface markers for further characterization [20]. The prognostic role of CTCs, for patients treated with radiotherapy and CRT, has been demonstrated [19,21,22], but its role in CRT-treated locally-advanced, metastatic or refractory ESCC is unclear. In this current study, CTC enumeration was achieved by a microfluidic chip. Viable CTCs were enriched with ultrafast speed by inertial force, depleting the vast majority of smaller-size white blood cells [23,24]. We examined the potential application of CTCs prospectively in longitudinal monitoring of patients for disease relapse or earlier detection of minimal residual disease (MRD). We have also explored the possibility of utilizing CTCs as a predictive biomarker for treatment response. Increasing recent evidence suggests the prognostic value of circulating cell-free DNA (cfDNA) for personalized monitoring of disease progression due to its good correlation with disease burden in various epithelial cancers [9,15,25,26,27,28]. Since it is convenient to collect blood samples non-invasively and analyze cfDNA and CTC simultaneously, the current study also explored the potential clinical application of combined changes of cfDNA and CTC in locally-advanced, metastatic, and refractory ESCC. The independent prognostic role of CTC counts and cfDNA levels at baseline, at the end of the second and the fourth cycles of chemotherapy, at treatment completion and relapse with interim response, disease progression and survival were examined. Our preliminary data suggested risk stratification of CT treatment outcomes and overall survival could be achieved based on the changes of baseline and pre-cycle III CTC and cfDNA, alone or combination. 

## 2. Results

### 2.1. Patient Characteristics

A total of 57 locally advanced stage III (25%) or metastatic stage IV (74%) ESCC patients receiving palliative CT were recruited for this study. The clinical information of these patients is summarized in Table 1. Most patients were male (86%) and their median age was 63, with a range from 49–76. There were 60% moderately differentiated and 39% poorly differentiated ESCC cases. The location of tumors at the upper, middle and lower positions of the esophagus are 27%, 42%, and 31%, respectively. The time-to-progression (TTP) is defined as the time of disease progression from the baseline CTC blood collection during interim reassessment without considering death as events. The progression-free survival (PFS) time is defined as the time of disease progression or death from the baseline CTC blood collection. The overall survival (OS) is defined as the time from the baseline CTC blood collection until death. The median TTP at interim reassessment was 74.5 days (range 19 to 122 days); 56% (29/52) patients had disease progression at interim reassessment. The median PFS time was 94 days (range 40–689 days); 91% (49/54) patients had disease progression or death. The median follow-up period was 181 days (minimum 40 days, maximum 1099 days) with 70% (40/57) deaths. Distant metastasis was present in 73.7% (42/57) ESCC patients at the time of baseline blood collection. Thirty-six and 64% of these 42 metastatic patients were synchronous and metachronous, respectively. About half (47%) of the patients were treatment-naïve. For the 30 patients receiving previous treatment, when the baseline CTC bloods were drawn, 19, 18 and 23 were treated with surgery, radiotherapy, or systemic therapy, respectively. Patients mainly received platinum-based chemotherapy regimens or a multimodality approach, combined with targeted or pathway-specific agents such as cetuximab or immunotherapy including nivolumab or pembrolizumab. Among the 23 patients with systemic therapy, 26.3% (15/57), 12.3% (7/57), 1.8% (1/57) received one, two and three regimens, respectively. The most frequent distant metastasis sites were lymph nodes (58%), liver (21%), and lung (23%). There was no statistically significant difference of baseline and pre-cycle III CTC counts or cfDNA levels between different clinical parameters, including median age, gender, stage at CTC blood sampling, grade, location of tumor, previous treatment, and primary tumor resection at the time of CTC blood drawing, and metachronous/synchronous metastasis. The relationships of CTC counts, cell-free DNA (cfDNA) levels, and clinical parameters are shown in Table 1. The pre-cycle III cfDNA level is statistically higher in the 12 patients without lymph node metastasis (3.627 ± 0.48) versus the 29 patients with lymph node metastasis (3.331 ± 0.32) (*t* test, *p* = 0.027). 

### 2.2. Role of CTC Enumeration for Prediction and Prognosis of Interim Reassessment, Progression-Free and Overall- Survival

#### 2.2.1. Serial CTC Enumeration 

**Figure 1:**
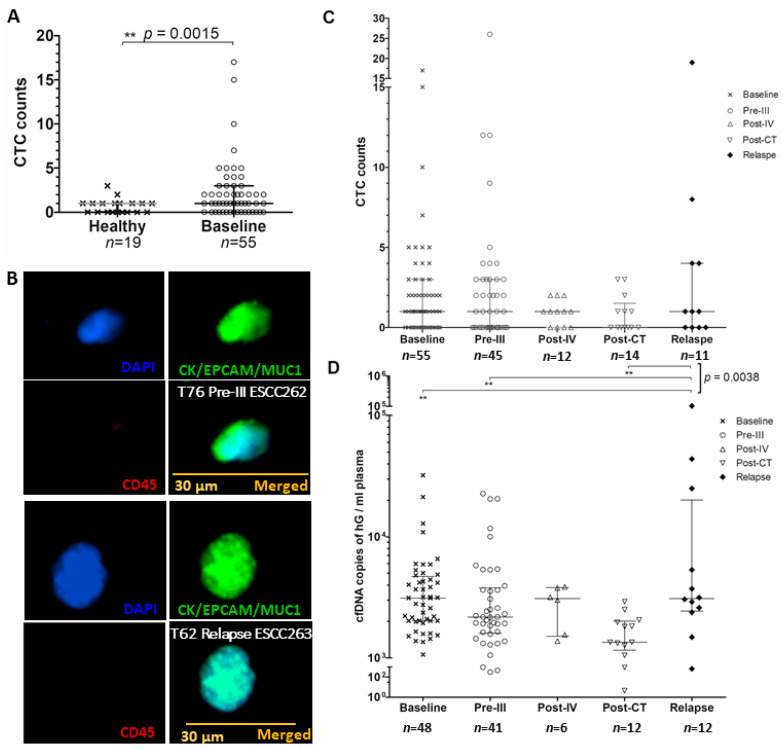
(**A**) CTC counts of healthy individuals and ESCC patients. (**B**) Representative CTC images from two ESCC patients. Liquid biopsy longitudinal serial monitoring of ESCC patents by (**C**) CTC counts and (**D**) cfDNA level. The median with interquartile range is indicated by error bar. The cfDNA levels were compared by ANOVA. ** *p* < 0.01.

Bloods were collected at 5 different time-points, as shown in Supplementary Figure S1. Baseline (CTC1), pre-cycle III (CTC2), post-cycle IV (CTC3), end of CT (CTC4), and relapse (CTC5) enumeration data were obtained for 55, 45, 12, 14, and 11 advanced ESCC patients, respectively. The mean, median, and other statistical details of CTC1, CTC2, CTC3, CTC4 and CTC5 counts are summarized in Supplementary Table S1. There is a statistically significant difference of CTC counts between 19 healthy individuals and 55 ESCC patients (Figure 1A) (*t* test, *p* = 0.0015). In the receiver operating characteristic (ROC) curve analysis of 45 ESCC patients and 19 healthy individuals (AUROC 0.681, *p* = 0.004), the baseline CTC test has a good specificity of 89.5% and a sensitivity of 45.5% (cut-off value = 1.5). Figure 1B shows representative CTC images from two ESCC patients receiving palliative CT collected at pre-cycle III and relapse. CTC counts at CTC1-CTC5 are summarized in Figure 1C. Median CTC counts at CTC1, CTC2, CTC3, and CTC5 are 1 CTC/5 mL and 0 CTC/5 mL for end of treatment CTC4. The ranges of CTC1-CTC5 counts are 17, 26, 2, 3, and 19 CTCs/5 mL, respectively. There was no statistical difference of CTC counts detected among CTC1-CTC5. Frequencies of patients with detectable CTCs at CTC1-CTC5 are 70.9% (39/55), 55.6% (25/45), 66.7% (8/12), 42.9% (6/14), and 54.5% (6/11), respectively. The decreased number of patients at CTC2 from CTC1 was partly attributed to patient withdrawal. The causes for patient withdrawal were due to side-effects of CT and repeated sampling. Failure for some cases was due to technical issue, when white blood cell counts were too high to be analyzed by our immunofluorescence (IF) enumeration protocol. The number of patients at CTC3-CTC5 dropped dramatically due to early disease relapses. Progression of disease occurred in 30 patients at interim reassessment. 

#### 2.2.2. Baseline CTC Enumeration Is Not Associated with Interim Reassessment, Progression-Free and Overall- Survival

The mean CTC1 and CTC2 enumerations were 2.31 and 2.47 cells/5 mL blood, respectively. Hence, the threshold of ≥3 CTCs was chosen, after considering the higher specificity of CTC1 and CTC2 for dichotomous high and low CTC groups for Kaplan-Meier and COX regression analysis. Baseline CTC count (≥3 CTCs vs. 0–2 CTCs) was not associated with interim reassessment, PFS, or OS (Table 2). However, a trend of association with response at interim reassessment was observed in these patients [hazard ratio (HR) 2.072, 95% CI 0.87–4.95, *p* = 0.101] (Table 2A). Kaplan-Meier survival analysis showed a trend of shorter progression time by interim imaging reassessment for patients with at least three CTCs (median TTP 79 days) at baseline compared to 0–2 CTC (median TTP 94 days) (*p* = 0.090) (Figure S2A).

#### 2.2.3. CTC Enumeration at Pre-Cycle III Chemotherapy Is Associated with Adverse Outcome

**Figure 2:**
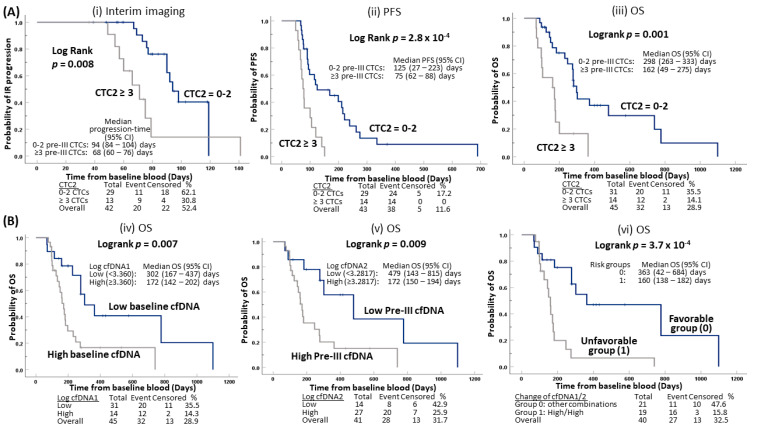
Kaplan-Meier survival analysis of (**A**) CTC enumeration at the end of two cycles of CT and (**B**) cfDNA at baseline, end of two cycles of CT, or their unfavorable change in advanced or refractory ESCC patients.

With the aim of determining whether CTC counts, taken at the end of two chemotherapy cycles, is an early predictive biomarker of treatment outcome, enumeration of CTC at pre-cycle III (CTC2) was obtained for 45 patients. Patients with at least three CTCs, i.e., the high CTC group, at the end of cycle II had significantly higher risk of progression at interim reassessment (HR 3.426, 95% CI 1.32–8.87, *p* = 0.011), worse PFS (HR 3.680, 95% CI 1.73–7.81, *p* = 0.001), and OS (HR 3.576, 95% CI 1.63–7.84, *p* = 0.001) compared to those with 0–2 CTCs (the low CTC group, Table 2). Furthermore, patients categorized as high CTC group (at least 3 CTCs, median TTP 68 days) at CTC2 experienced statistically significant shorter time of progression evaluated by imaging reassessment compared to those of the low CTC group (0–2 CTCs, median TTP 94 days) (Figure 2Ai). The median PFS of patients of the high CTC group at CTC2 (median PFS 75 days) was statistically significantly shorter compared to those of the low CTC group (median PFS 125 days) (Figure 2Aii). The median OS of patients from the high CTC group at CTC2 (median OS 162 days) was statistically significantly shorter compared to those from the low CTC group (median OS 298 days) (Figure 2Aiii). 

### 2.3. Role of cfDNA Levels for Prediction and Prognosis of Interim Reassessment, Progression-Free and Overall- Survival

#### 2.3.1. Serial cfDNA Levels

The amounts of cfDNA obtained at baseline (cfDNA1), pre-cycle III (cfDNA2), post-cycle IV (cfDNA3), end of CT (cfDNA4) and relapse (cfDNA5) are summarized in Figure 1D. The mean, median, standard deviation, range, quartiles, minimum and maximum levels of cfDNAs are detailed in Supplementary Table S1. The median cfDNA levels (standard deviation) at cfDNA1, cfDNA2, cfDNA3, cfDNA4 and cfDNA5 are 3123 (5348), 2176 (5368), 3092 (1085), 1579 (600) and 3095 (30,205) copies of haploid genome (hG)/mL plasma, respectively. The cfDNA levels ranged from 31,169, 22,391, 2487, 2000, and 102,537 copies of hG/mL plasma, respectively. The cfDNA level in copies of hG/mL plasma for patients at the relapse was significantly higher than those at the baseline, pre-cycle III or post-CT (Figure 1D). 

#### 2.3.2. Baseline and CT Pre-Cycle III cfDNA Levels Are Associated with Overall Survival

Baseline (cfDNA1) and pre-cycle III (cfDNA2) cfDNA were obtained for 48 and 41 patients, respectively. COX regression analysis utilizing cfDNA1 and cfDNA2 levels as a continuous variable after log10 transformation were not significantly associated with interim imaging reassessment and PFS despite a trend of association with interim reassessment observed in patients with higher baseline cfDNA level (HR 4.980, 95% CI 0.91–27.22, *p* = 0.064, Table 2A). However, levels of cfDNA1 (HR 8.338, 95% CI 2.42–28.7, *p* = 0.001) and cfDNA2 (HR 5.451, 95% CI 1.74–17.1, *p* = 0.004) are significantly associated with OS (Table 2C). Based on Receiver operating characteristic (ROC) analysis, the thresholds of baseline and pre-cycle III log cfDNA levels were chosen at 3.360 (~2291 copies in hG/mL plasma) and 3.2817 (~1913 copies in hG/mL plasma) for OS, after considering sensitivity and specificity (Table S2). The patients were dichotomized into high and low cfDNA level groups, and their Kaplan-Meier curves were compared for OS (Figure 2B). Patients with high baseline cfDNA1 (median OS 172 days) had a statistically significant shorter OS time compared to those with low baseline cfDNA1 (median OS 302 days) (*p* = 0.007) (Figure 2Biv). Patients with high cfDNA2 at pre-cycle III (median OS 172 days) had a statistically significant shorter OS time compared to those with low cfDNA2 (median OS 479 days) (*p* = 0.009) (Figure 2Bv). Patients were divided into favorable and unfavorable risk groups considering their changes of cfDNA1 and cfDNA2. Patients with unfavorable changes of both high cfDNA1 (≥3.360) and cfDNA2 (≥3.2817) are categorized into the high-risk group, while others including those with both low cfDNA1 and cfDNA2, or from high cfDNA1 to low cfDNA2, or with low cfDNA1 and high cfDNA2 are categorized into the low-risk group. The favorable group of patients (median OS 363 days) had a statistically significant longer OS time compared to those with unfavorable change of baseline to pre-cycle III cfDNA levels (median OS 160 days) (*p* = 3.7 × 10^−4^) (Figure 2Bvi).

### 2.4. Early Prediction of Disease Progression for Patients with Unfavorable Changes of CTC Counts from Baseline to Pre-Cycle III 

**Figure 3:**
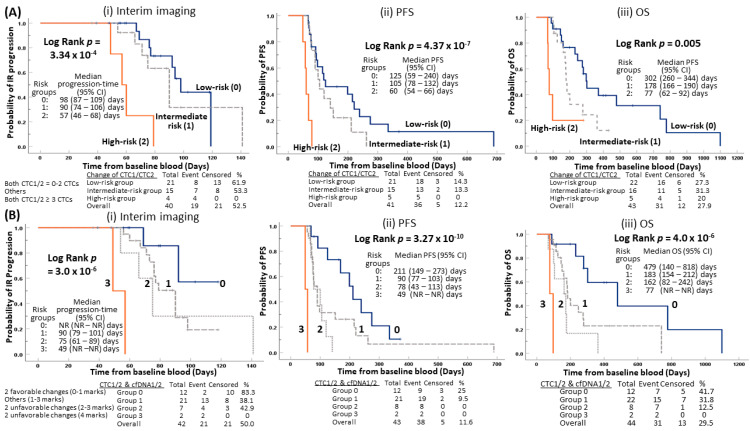
Risk stratification of disease progression. Change of CTC1/CTC2 (**A**), combined changes of CTC and cfDNA (**B**) were associated with interim reassessment imaging, PFS, and OS.

We examined the predictive value of change in CTC counts from baseline to pre-cycle III. Patients with favorable changes of both low baseline and pre-cycle III CTC (0–2 CTCs) were considered the low-risk group (0). Patients with unfavorable changes of both high baseline and pre-cycle III CTC (≥3 CTCs) were considered as the high-risk group (2). Patients having other CTC1/CTC2 changes were considered as the intermediate-risk group (1), including changes from low baseline CTC to high pre-cycle III CTC and high baseline CTC to low pre-cycle III CTC. The low-risk group patients (median TTP 98 days) experienced statistically significant longer time of progression evaluated by imaging reassessment compared to the intermediate-risk group (median TTP 90 days) and the high-risk group (median TTP 57 days) (*p* = 3.34 × 10^−4^) (Figure 3Ai). The low-risk group patients (median PFS 125 days) experienced statistically significant longer PFS, compared to intermediate-risk group (median PFS 105 days) and the high-risk group (median PFS 60 days) (*p* = 4.37 × 10^−7^) (Figure 3Aii). The low-risk group patients (median OS 302 days) had statistically significant longer survival times compared to the intermediate-risk group (median OS 178 days) and the high-risk group (median OS 77 days) (*p* = 0.005) (Figure 3Aiii).

### 2.5. Early Prediction for Disease Relapse for Patients with Integration of Unfavorable Changes of CTC Counts and cfDNAs from Baseline to Pre-Cycle III 

Improvement of risk stratification, based on the combined change of CTC and cfDNA for the disease outcome, was compared to that indicated solely based on the change of baseline CTC and pre-cycle III CTC shown in the above Section 2.4 (Figure 3A). Hence, we further explored risk stratification of patients into four risk groups, based on integration of changes of both CTC1/CTC2 and cfDNA1/cfDNA2 levels from baseline to pre-cycle III (Figure 3B). The definition of risk groups is provided in Table S3. Each specimen from CTC1, CTC2, cfDNA1, and cfDNA2 was categorized into high (1 mark) and low (0 mark) groups. Patients with two favorable changes of 0–1 mark, combining CTC1/CTC2 and cfDNA1/cfDNA2, were designated as the low-risk group (0). Patients with two unfavorable changes of 4 marks were considered as the high-risk group (3). Patients with two unfavorable changes of 2–3 marks, combining CTC and cfDNA changes, were considered as the at- risk group (2). Other combinations included patients having one unfavorable change and/or one favorable change with 1–3 marks, either from CTC or cfDNA changes, were considered as the at-risk group (1). The group 0 patients (median TTP NR days) experienced statistically significant longer times of progression evaluated by imaging reassessment compared to the at-risk group 1 (median-progression 90 days), at-risk group 2 (median TTP 75 days) and the highest risk group 3 (median TTP 49 days) (*p* = 3.0 × 10^−6^) (Figure 3Bi). The lowest risk group 0 patients (median PFS 211 days) experienced statistically significant longer PFS compared to group 1 (median PFS 90 days), group 2 (median PFS 78 days), and the highest risk group 3 (median PFS 49 days) (*p* = 3.27 × 10^−10^) (Figure 3Bii). Similarly, the group 0 patients (median OS 479 days) experienced statistically significant longer survival times compared to group 1 (median OS 183 days), group 2 (median OS 162 days), and the group 3 (median OS 77 days) (*p* = 4 × 10^−6^) (Figure 3Biii).

### 2.6. COX Regression Analysis of Independent Prognostic Role of CTC and cfDNA Values

In order to achieve the primary objective of identification of early predictive biomarkers for treatment outcome of advanced ESCC patients, various clinical variables, baseline and pre-cycle III CTC counts and cfDNA levels were analyzed by the univariate and multivariate COX regression models for interim reassessment, PFS, and OS (Table 2). Interim reassessment progression was significantly associated with stage at CTC blood sampling, lymph node (LN) metastasis, and pre-cycle III CTC counts by univariate COX regression analysis. In multivariate regression analysis, only pre-cycle III CTC remained as an independent prognostic factor for interim reassessment by forward stepwise analysis (Likelihood Ratio) (HR 3.426, 95% CI 1.32–8.87, *p* = 0.011) (Table 2A). PFS was significantly associated with the stage and at primary tumor resection being performed when baseline CTC blood samples were taken, liver metastasis, and pre-cycle III CTC counts by univariate COX analysis (*p* < 0.05). In the multivariate regression analysis with forward stepwise (Likelihood Ratio), a total of four variables, were also included. The CTC counts taken at pre-cycle III (HR 4.014, 95% CI 1.81–8.88, *p* = 0.001) and primary tumor resection (HR 0.402, 95% CI 0.19–0.87, *p* = 0.02) remained the independent prognostic factors for PFS (Table 2B). For OS, univariate COX analysis showed significant association with the age, pre-cycle III CTC count, baseline and pre-cycle III cfDNA levels, change of cfDNA1/2, change of CTC1/2, and the combined changes of baseline and pre-cycle III CTC counts and cfDNA level (CTC1/2 and cfDNA1/2). A Spearman’s rank-order correlation showed that there were statistically significant correlations between cfDNA1 and cfDNA2, the combined change of CTC1/2 and cfDNA1/2 with CTC2, cfDNA1, cfDNA2, the change of cfDNA1/2, and the change of CTC1/2 (Supplementary Table S4). The multivariate COX regression analysis with forward stepwise (Likelihood Ratio), indicated that both age (HR 0.932, 95% CI 0.87–0.99, *p* = 0.032) and the combined changes of CTC1/2 and cfDNA1/2 (HR_group1_ 6.008, 95% CI 1.27–28.5, HR_group2_ 9.520, 95% CI 1.81–50.0, HR_group3_ 81.958, 95% CI 7.95–845, *p* = 0.002) remained independent prognostic factors for OS (Table 2C). 

## 3. Discussion

Locoregionally advanced and metastatic ESCC is deadly, with a median OS ranging from 7–10 months after treatment by the standard 5-fluorouracil (5-FU) and cisplatin-based first-line palliative CT [29]. There is an unmet clinical need to identify early predictive biomarkers of treatment response of these advanced ESCC patients for timely identification of chemotherapy resistance, and thus, guidance for optimizing treatment strategy. Our current prospective longitudinal analysis of liquid biopsy in patients with advanced ESCC, treated by CT or CTRT suggested CTC and cfDNA real-time serial monitoring, provides useful complementary predictive and prognostic information for CT response and overall survival. The clinical utility of CTCs in ESCC was reported by various molecular detection techniques including mRNA and Q-PCR assay and depletion of white blood cells by CD45 magnetic beads [21,30,31]. A previous study in ESCC reported CTC enumeration by Cell-Search^®^ before and after chemotherapy or chemoradiation therapy is a promising predictive biomarker [19]. However, this previous ESCC studies using EpCAM-based CTC isolation strategy may underestimate the highly heterogeneous CTC population, and might miss those aggressive refractory CTCs undergoing EMT or CTCs, with cancer stemness properties [8,17]. Hence, the current study utilized a size-based microfluidic depletion strategy to enrich for CTCs after centrifugal force removal of smaller leucocytes. In the current prospective study, a higher positive baseline and pre-cycle III CTC counts were detected in 70.9% (39/55), and 55.6% (25/45) of ESCC patients, respectively [19]. Multivariate COX analysis suggested that CTC counts obtained six weeks after two cycles of CT (CTC2) was the only independent factor associated with early-disease progression (HR ~3.7) at interim reassessment taken post-cycle IV at week 10 (Table 2A). For PFS, our results suggest that both high pre-cycle III CTC counts (HR~4) and patients without primary tumor resection were independent prognostic factors of treatment failure (Table 2B). The current study provides the first evidence to suggest the use of pre-cycle III CTC enumeration to contribute important predictive information, and help physicians decide on early switching to next line of therapy.

We observed a trend of association between early-disease progression, determined by interim imaging reassessment with high baseline CTC count in the univariate analysis (Table 2A), and Kaplan-Meier survival analysis (*p* = 0.090) (Figure S2A). Although, it was not statistically significant. However, stratification of the 26 patients without previous treatment with high baseline CTC counts (median TTP 61 days) revealed a statistically significant shorter progression time for clinical interim reassessment for patients with low baseline CTC counts (median TTP 94 days) (*p* = 0.037) (Figure S2B); no such significant difference was observed for the group of 24 patients with previous treatment (Figure S2C). The prognostic role of baseline CTC counts requires further validation in a larger sample size of previously untreated advanced ESCC. 

Earlier cfDNA analysis includes heterogeneous groups of esophageal cancers, comprising both adenocarcinoma and ESCC, and other gastrointestinal cancers, but only a few of these focused explicitly on advanced ESCC, as in our current study [25,27,32,33,34,35,36]. The clinical utility of higher cfDNA level or detection of copy number variants (CNVs) or single nucleotide variants (CNVs) are predictive and useful for stratification of esophageal cancer outcomes of patients into both early-stage and advanced cohorts [25,32,33,36]. Previous EC cfDNA analysis focused on pre-treated or post treatment levels. Our current prospective study additionally examined the prognostic value of pre-cycle III and post-cycle IV time-points. The majority of our patients progressed during treatment and only 12 patients (21%, 12/57) completed the CT treatment. The higher cfDNA level (≥log cfDNA5 3.4384, ~2744 copies of hG/mL plasma), obtained at relapse for this subset of 12 patients showed a trend of association with shorter OS compared to patients with lower cfDNA level (Log Rank *p* = 0.12, data not shown). A similar trend was observed for CTC5 for patients with positive versus negative CTC status (Log Rank *p* = 0.068), but these differences of cfDNA5 and CTC5 were not statistically significant. We observed that patients with high levels of pre-treated cfDNA or pre-cycle III or unfavorable changes from baseline to pre-cycle III cfDNA have significantly higher risk of death in locally advanced and metastatic ESCC (Figure 2B). The approach using a combination of different parameters has been proposed previously, such as the prognostic utility of integration of cfDNA with imaging-based response for early-stage EC [25]. Our earlier study in nasopharyngeal carcinoma (NPC) reported the clinical utility of integration of both EBV plasma DNA and CTC analysis for complementary enhancement of earlier detection of MRD in metastatic NPC patients with complete response by imaging [28]. To the best of our knowledge, there are no integration studies for serial monitoring of both the CTC enumeration and cfDNA level to examine its prognostic role. Compared with CTCs, which are very rare with an estimated frequency of 1 CTC in 10^6^–10^8^ white blood cells, cfDNA is more stable and technically easier to isolate than CTCs, and has a higher sensitivity and wider dynamic range. CTCs are intact cells shed from the primary or metastatic tumor sites, while cfDNA is nucleic acid that is shed into the bloodstream and is linked to apoptotic or necrotic cancer cells [37]. Thus, CTCs may be more representative of the tumors. They carry different but supplementary information underlying tumor growth kinetics, drug resistance and tumor metastasis. cfDNA is a good biomarker of tumor burden for detection of minimal residual tumor. CTCs are considered as the metastatic seeds, which are highly invasive, resistant to apoptosis and anoikis in the hostile blood circulation environment, and with the plasticity to evolve drug resistance and colonize the distant lesion. The sub-populations of CTCs possess the cancer stem cell-like properties and epithelial-to-mesenchymal transition (EMT)+ associated phenotypes, like drug resistance and cancer dissemination [9,15,17]. The cfDNA and CTC are synergistic and independent markers. In the multivariable Cox analysis, both changes of CTC counts and cfDNA levels from baseline to pre-cycle III were significantly associated with OS and PFS after adjusted for age and stage (Table S5). There are also recent literature reviews suggesting the use of the combination of both CTCs and cfDNA as complementary biomarkers [9,15]. The combination of CTC and cfDNA level is convenient to the patient as only one blood taking is required for multi-parametric analysis. Here, we first report that risk stratification of patients into four groups, according to their combined status of unfavorable and favorable changes, at baseline and pre-cycle III CTC1/2, and cfDNA1/2 being an independent prognostic marker for OS in locally advanced and metastatic ESCC patients (Table 2C). The risk stratification, based on CTC change analysis alone, is already very useful without identification of cfDNA level. However, future characterization of specific mutation profiles in cfDNA might be added to improve personalization of the risk stratification of treatment outcome.

Generalization of the results of our study are limited, due to the relatively modest cohort size and patients being recruited after previous treatment. However, multivariate COX analysis suggested the pre-cycle III CTC enumeration remained an independent prognostic factor for both, interim reassessment and PFS. The power of this study remains statistically strong even with the current sample size. For interim reassessment, the power of this pre-cycle III CTC enumeration study was 97.7% with the sample size of 42 and disease progression occurring in 0.56 proportion of patients with observed HR = 3.426, and α set at 0.05. For PFS, the power of the pre-cycle III enumeration study was 75% with the sample size of 43; disease progression occurred in 0.907 proportion of patients with observed HR = 4.014, and α set at 0.05. The potential clinical utility of integration of the unfavorable CTC and cfDNA status at baseline and pre-cycle III for OS was limited by the high-risk group 3, which has a small sample size (*n* = 2 in Figure 3Biii), and hence, further validation studies are warranted. Similarly, preliminary data from further risk stratification that high-risk group 3 (*n* = 4 in Figure 3Ai, *n* = 5 in Figure 3Aii–iii), defined by unfavorable high baseline and high pre-cycle III CTC counts, and integrated changes of both CTC1/2 and cfDNA1/2 levels (Figure 3Bi–ii), had the shortest time of disease progression during interim reassessment, PFS and OS should be considered as exploratory and hypothesis-generating only. The major study limitation is the small sample size, especially with respect to follow-up samples. Future validation studies with independent cohorts are necessary to verify the potential clinical utility of changes of liquid biopsy status, at baseline and pre-cycle III, for prediction of early-disease progression. Currently, CTC and cfDNA data were treated as independent time-points in the conventional COX regression analysis. A joint model may be useful to identify the ideal follow-up time-point in the future studies with a larger patient cohort and complete data for longitudinal analysis. Another limitation of the current CTC assay may be attributed to the utilized isolation strategy to efficiently capture CTCs of small size. However, our size separation strategy enables the isolation of viable CTCs for further culture and characterization. The antibody-independent capture strategy is used to explore the heterogenous CTC populations not limited to epithelial plasticity in CTCs. The role of EMT in cancer, particularly in metastasis, is complex and under active debate [8,38]. CTC enumeration with various markers should be used in order to identify and better characterize the other populations of CTCs undergoing an EMT or with intermediate phenotype of partial EMT, and with stem cell-like properties [39]. Improvements in CTC characterizations to gain mechanistic knowledge in the metastasis cascade, regarding EMT plasticity, cancer stem cell-like properties, and the role of immune cells in initiation of metastatic dissemination of carcinoma cells are expected to benefit patients by translation in clinical utility. Since our study only quantified the total amount of cfDNA without detection of specific mutant variants, we cannot rule out the possibility that an increase of cfDNA indicates non-specific tissue side reactions and/or inflammation. We expect future liquid biopsy real-time monitoring studies should tackle the specificity and sensitivity issues of the current assays with technological enhancement from the next-generation sequencing and transcriptomic approaches. Implementation of serial monitoring CTC and cfDNA analysis for detection of minimal residual disease and stratification of patients for the risk of disease relapse or early prediction of treatment outcome is needed for precise personalized medicine. 

## 4. Materials and Methods 

### 4.1. Study Population

A total of 60 locally advanced or metastatic ESCC patients receiving palliative CT were recruited from 2016–2019 at Queen Mary Hospital. Supplementary Figure S1 illustrates the timeline for blood specimen collection sampling. During the palliative chemotherapy (CT) for six cycles, baseline blood was taken before treatment (CTC1), after two cycles of CT (CTC2), after four cycles of CT (CTC3), at the end of CT (CTC4) and at relapse (CTC5) to correlate with the interim and final Positron Emission Tomography-Computed Tomography (PET/CT) imaging, respectively. Guidelines from the European Organisation for Research and Treatment of Cancer (EORTC) were followed to define tumor response by PET-CT imaging. Complete metabolic response (CMR) is defined as complete resolution of FDG uptake in all lesions, partial metabolic response (PMR) has ≥25% reduction in the sum of SUVmax after more than one cycle of treatment, and progressive metabolic disease (PMD) is defined as having ≥25% increase in the sum of SUVmax or appearance of new FDG-avid lesions. Stable metabolic disease (SMD) is defined as being neither CMR, PMR, nor PMD. A total of 143 blood samples were collected from these 60 patients. Three patients were excluded due to refusal of treatment or death before treatment. Among the 57 remaining, eight patients were recruited twice for two lines of palliative treatment. Informed consent for sample collection from the ESCC patients was obtained according to protocols approved by the Institutional Review Board (IRB) of the University of Hong Kong/Hospital Authority Hong Kong West Cluster (HKU/HA HKW IRB) (ethic code:MLLMETGI2017). The study was performed in accordance with the Declaration of Helsinki.

### 4.2. CTC Enrichment and Enumeration

STRECK tubes were used for collection of 5 mL peripheral blood and processed within 72 h. The size-based CTC enrichment utilized CTChip^®^FR1 microfluidic chips performed on a ClearCell^®^FX1 System from Biolidics (Singapore) and immunofluorescence enumeration procedures were performed as previously described [28,40]. In brief, after red blood cell lysis, white blood cells (WBCs) were centrifuged at 500× *g* at room temperature for 10 min. The WBC pellet was gently but thoroughly resuspended and subjected to inertial centrifugal force to separate smaller WBCs [23,24]. Output cells were collected and fixed on a poly-L-lysine slide and stained with pan-CK/EpCAM/MUC1-Alexa 488 conjugated (Pan-Keratin C11, Cell Signaling; Pan-Cytokeratin AE1/AE3, eBioscience; EpCAM VU1D9, Cell Signaling; CD227/Mucin1 SM3, eBioscience) and CD45-APC conjugated (BD Pharmingen) antibodies [28]. The cell images, obtained from the ESCC serial blood samples, were captured by Cytation 5 Cell Imaging Multi-Mode Reader (BioTeck, USA). The imaged CTCs were enumerated by imaging softwares CellProfiler and CellProfiler Analyst and R script to identify the potential CTCs and then subsequently confirmed by manual inspection [28,40]. Cells staining positive for DAPI and pan-CK/EpCAM/MUC1 and negative for CD45 are considered CTCs.

### 4.3. cfDNA Isolation and Quantification

Plasma was isolated from whole blood samples by centrifuging at 500× *g* for 10 min and stored at −80 °C until further use. The cfDNA was extracted from 2–5 mL plasma using the AVENIO cfDNA isolation kit (Roche Diagnostics), according to manufacturer’s instruction. In brief, the plasma sample was spun at 1800× *g* for 5 min to remove precipitates and debris, followed by proteinase K digestion. After the incubation with DNA Paraffin Binding Buffer (PBB) and isopropanol, the cfDNA was transferred to the High Pure Extender Assembly (HPEA) and the cfDNA was captured by the filter tube inside the HPEA. cfDNA was washed twice and eluted with DNA Elution Buffer, and then stored in −20 °C until use. cfDNA was quantified using a Qubit fluorometer 2.0 (Invitrogen Ltd., Life Technologies, USA) and a Qubit dsDNA HS Assay Kit (Invitrogen Ltd., Thermo Fisher Scientific), according to manufacturer’s instructions.

### 4.4. Statistical Analysis

Chi square and Fisher’s exact tests were used for comparison between CTC status and categorical clinicopathological factors. The cfDNA levels were log10-transformed and included as continuous variables. Comparison between log cfDNA level and categorical clinicopathological factors was done by *t*-test. The CTC status or log cfDNA level were classified into high or low for comparison of survival. Kaplan-Meier analysis was used to estimate disease progression of interim reassessment, PFS and OS. Univariable and multivariate Cox proportional hazards models were used for examination of association of various clinical parameters, baseline and pre-cycle III CTC counts (CTC1 and CTC2), and baseline and pre-cycle III cfDNA level (cfDNA1 and cfDNA2), and their changes from baseline to pre-cycle III, alone or combined. The cfDNA level was log_10_-transformed and included as a continuous variable. *p* values <0.05 were considered as statistically significant. Analyses were performed with SPSS v26 (SPSS Inc, IBM Corporation, Armonk, NY, USA). 

## 5. Conclusions

We prospectively followed the change in CTC and cfDNA in patients with advanced ESCC treated by palliative CT. This study shows that the CTC count at pre-cycle III is independently associated with interim reassessment and PFS. Combined changes of CTC count and cfDNA level status from baseline to pre-cycle III are independently associated with OS. Baseline and pre-cycle III liquid biopsy analysis can identify patients with advanced ESCC at increased risk of early-disease progression, treatment failure and death. Our preliminary data leads to the hypothesis that longitudinal liquid biopsy serial monitoring, when used complementarily, provide predictive and prognostic information for CT response and death in advanced ESCC. Future larger studies in independent cohorts will be required to validate the predictive and prognostic value observed for the clinical utility of early switching to next-line therapies.

## Figures and Tables

**Table 1:** Baseline characteristics of CTC bloods for CTC counts and cfDNA levels of ESCC patients.

Parameters	Patients (*n* = 57)	CTC1		CTC2		cfDNA1 ^d^ Median ± SD	cfDNA2 ^d^ Median ± SD
		≥3	0–2	≥3	0–2		
**Median age (range)**	63 (49–76)						
<63	28 (49%)	6	22	7	15	3425 ± 2496	2311 ± 3231
≥63	29 (51%)	9	18	7	16	2577 ± 6914	2171 ± 6482
Sex							
Male	49 (86%)	13	34	12	27	3116 ± 3690	2176 ± 4665
Female	8 (14%)	2	6	2	4	4186 ± 11004	2755 ± 8598
**G category ^a^**							
GX	1 (1.8%)	-		-		-	-
G2	34 (59.6%)	8	24	8	20	2663 ± 3975	2176 ± 5316
G3	22 (38.6%)	6	16	6	11	3682 ± 7054	2065 ± 5616
**Tumor location**							
Upper	9 (27.3%)	4	4	3	5	2580 ± 1719	2447 ± 1326
Middle	26 (41.8%)	5	21	6	12	3171 ± 2201	2167 ± 3283
Lower	19 (30.9%)	5	13	5	13	2220 ± 8927	1939 ± 6789
Unknown	3 (5.3%)	-		-		-	-
**Stage ^b^**							
III	14 (24.6%)	5	9	4	5	2111 ± 6785	3598 ± 7188
IV	42 (73.7%)	10	30	10	25	3137 ± 5113	2167 ± 5054
Unknown	1 (1.8%)	-		-		-	-
**Previous treatment**							
No	27 (47.4%)	7	20	6	12	3171 ± 4662	3069 ± 6395
Yes	30 (52.6%)	8	20	8	19	3116 ± 5911	1954 ± 4513
**Distant Metastasis**							
No	15 (26.3%)	5	10	4	6	2111 ± 6785	3598 ± 7188
Yes	42 (73.7%)	10	30	10	25	3137 ± 5113	2167 ± 5054
Synchronous metastasis	15 (35.7%)	4	11	3	8	3679 ± 3077	2429 ± 6126
Metachronous metastasis	27 (64.3%)	6	19	7	17	2954 ± 6018	2048 ± 4650
Metastasis to one site	25 (43.9%)	8	16	4	14	3130 ± 6484	1954 ± 6432
Metastasis to ≥2 sites	17 (29.8%)	2	14	6	11	3143 ± 2142	2171 ± 2581
**Site of Metastasis**							
Liver	12 (21.1%)	1	10	3	9	3130 ± 2515	2176 ± 3063
Lung	13 (22.8%)	4	8	5	5	2791 ± 8211	2167 ± 8013
Lymph node	33 (57.9%)	6	25	7	22	3123 ± 1997	2048 ± 2550
Others ^c^	11 (19.4%)	-		-		-	-

^a^ Squamous cell carcinoma G category: GX, G2, G3 = Differentiation cannot be assessed, well, moderately, and poorly differentiated, respectively. ^b^ 7th edition of American Joint Committee on Cancer (AJCC) TNM system for Cancer Staging. ^c^ Others include adrenal (1), bone (6), pleura (2), peritoneum (1) and spleen (1). ^d^ Copies of hG/mL plasma.

**Table 2:** Univariate and multivariate COX regression analysis of clinical parameters, CTC counts and cfDNA levels at baseline and two cycles of chemotherapy (Pre-cycle III) with interim imaging reassessment, progression-free and overall survival.

Variables	Univariate Analysis		Multivariate Analysis ^b^	
	HR (95% CI)	*p*-Value	HR (95% CI)	*p*-Value
**(A) Interim Reassessment** (*n* = 52)				
Age	1.003 (0.95–1.06)	0.906	-	-
Gender (F vs. M ref)	1.553 (0.52–4.62)	0.428	-	-
L cat (Others vs. Lower ref)	0.886 (0.40–1.96)	0.766	-	-
G cat (G3 vs. G2 ref)	0.615 (0.29–1.41)	0.251	-	-
Previous treatment (Yes vs. no ref)	0.882 (0.40–1.93)	0.753	-	-
Stage at CTC blood collection (IV vs. III ref)	**0.262 (0.11–0.62)**	**0.002**	-	-
Primary tumor resected at CTC blood collection (*n* = 50) (Yes vs. no ref)	0.548 (0.22–1.34)	0.201	-	-
Metastasis Meta vs. Syn ref (*n* = 38)	1.310 (0.48–3.55)	0.596	-	-
LN metastasis (Yes vs. no ref)	**0.392 (0.18–0.84)**	**0.016**	-	-
Lung metastasis (Yes vs. no ref)	1.333 (0.54–3.32)	0.537	-	-
Liver metastasis (Yes vs. no ref)	0.715 (0.24–2.09)	0.541	-	-
Baseline CTC count (3 vs. 0–2 ref) (*n* = 50)	2.072 (0.87–4.95)	0.101	-	-
Pre-cycle III CTC count (3 vs. 0–2 ref) (*n* = 42)	**3.426 (1.32–8.87)**	**0.011**	**3.426** **(1.32–8.87)**	**0.011**
Baseline log cfDNA * (*n* = 39)	4.980 (0.91–27.22)	0.064	-	-
Pre-cycle III log cfDNA * (*n* = 35)	2.241 (0.77–6.53)	0.139	-	-
**(B) Progression-free survival (PFS)** (*n* = 54)				
Age	0.956 (0.91–1.00)	0.062	-	-
Gender (F vs. M ref)	0.969 (0.45–2.08)	0.935	-	-
L cat (Others vs. Lower ref) (*n* = 53)	1.286 (0.70–2.35)	0.414	-	-
G cat (G3 vs. G2 ref) (*n* = 53)	0.867 (0.48–1.56)	0.633	-	-
Previous Treatment (Yes vs. no ref)	0.561 (0.30–1.05)	0.071	-	-
Stage at CTC blood collection (IV vs. III ref)	**0.430 (0.22–0.85)**	**0.016**	-	-
Primary tumor resected at CTC blood collection (*n* = 51) (Yes vs. no ref)	**0.373 (0.19–0.75)**	**0.005**	**0.402** **(0.19–0.87)**	**0.02**
Metastasis (Meta vs. Syn ref) (*n* = 40)	0.517 (0.25–1.08)	0.080	-	-
LN metastasis (Yes vs. no ref)	0.618 (0.35–1.11)	0.106	-	-
Lung metastasis (Yes vs. no ref))	1.167 (0.60–2.25)	0.646	-	-
Liver metastasis (Yes vs. no ref)	**0.449 (0.21–0.98)**	**0.043**	-	-
Baseline CTC count (≥3 vs. 0–2 CTCs ref) (*n* = 52)	1.349 (0.69–2.63)	0.380	-	-
Pre-cycle III CTC count (≥3 vs. 0–2 CTCs ref) (*n* = 43)	**3.680 (1.73–7.81)**	**0.001**	**4.014** **(1.81–8.88)**	**0.001**
Baseline log cfDNA * (*n* = 47)	1.959 (0.67–5.76)	0.222	-	-
Pre-cycle III log cfDNA * (*n* = 40)	1.681 (0.70–4.06)	0.249	-	-
**(C) Overall survival (OS)** (*n* = 57)				
Age	**0.940 (0.89–0.99)**	**0.027**	**0.932** **(0.87–0.99)**	**0.032**
Gender (F vs. M ref)	0.886 (0.38–2.04)	0.776	-	-
L cat (Others vs. Lower ref) (*n* = 56)	1.884 (0.89–3.99)	0.097	-	-
G cat (G3 vs. G2 ref) (*n* = 53)	0.821 (0.43–1.57)	0.552	-	-
Previous Treatment (Yes vs. no ref)	0.534 (0.28–1.02)	0.056	-	-
Stage at CTC blood collection (IV vs. III ref) (*n* = 56)	0.620 (0.30–1.27)	0.192	-	-
Primary tumor resected at CTC blood drawn (*n* = 53) (Yes vs. no ref)	0.584 (0.29–1.17)	0.127	-	-
Metastasis (Meta vs. Syn ref) (*n* = 42)	0.565 (0.27–1.21)	0.140	-	-
LN metastasis (Yes vs. no ref)	0.926 (0.48–1.78)	0.821	-	-
Lung metastasis (Yes vs. no ref)	1.002 (0.46–2.20)	0.997	-	-
Liver metastasis (Yes vs. no ref)	0.784 (0.37–1.67)	0.529	-	-
Baseline CTC count (≥3 vs. 0–2 CTCs ref) (*n* = 55)	0.973 (0.44–2.14)	0.946	-	-
Pre-cycle III CTC count (≥3 vs. 0–2 CTCs ref) (*n* = 45)	**3.576 (1.63–7.84)**	**0.001**	-	-
Baseline log cfDNA * (*n* = 48)	**8.338 (2.42–28.7)**	**0.001**	-	-
Pre-cycle III log cfDNA * (*n* = 41)	**5.451 (1.74–17.1)**	**0.004**	-	-
Change of cfDNA1/2 (*n* = 40)			-	-
Group 0 (Other combinations, *n* = 21)	Reference			
Group 1 (High cfDNA1 & cfDNA2, *n* = 19)	**4.444 (1.85–10.68)**	**0.001**		
Change of CTC1/2 (*n* = 43)			-	-
Group 0 (Low CTC1 & CTC2, *n* = 22)	Reference	**0.012**		
Group 1 (Other combinations, *n* = 16)	3.103 (1.11–8.65)	0.089		
Group 2 (High CTC1 & CTC2, *n* = 5)	**6.178 (1.87–20.4)**	**0.004**		
Combined changes of CTC and cfDNA ^a^				
(*n* = 44)				
Group 0 (*n* = 12)	Reference	**0.001**		**0.002**
Group 1 (*n* = 22)	**3.103 (1.11–8.65)**	**0.030**	**6.008 (1.27–28.5)**	**0.008**
Group 2 (*n* = 8)	**6.178 (1.87–20.4)**	**0.030**	**9.520 (1.81–50.0)**	**0.008**
Group 3 (*n* = 2)	**53.07 (7.02–401)**	**1.2 × 10^−4^**	**81.958 (7.95–845)**	**2.2 × 10^−4^**

* Log_10_ transformed for cfDNA (copies of hG/mL of plasma) as a continuous variable. ^a^ Group 0–Two favorable changes with 0–1 mark; Group 1–One favorable change with 1–3 marks; Group 2–Two unfavorable changes with 3 marks; Group 3–Two unfavorable changes with 4 marks. One mark is given for positive status of CTC1, CTC2, log cfDNA1, and cfDNA2 amount. Positive CTC1 and CTC2 are ≥3 CTCs, positive log cfDNA1 is ≥3.360, positive log cfDNA2 is ≥3.2817. ^b^ Multivariate COX regression model for interim reassessment with forward stepwise (Likelihood Ratio) including stage at CTC blood drawn, lymph node metastasis, pre-cycle III CTC counts. ^b^ Multivariate COX regression model for PFS with forward stepwise (Likelihood Ratio), including the stage at CTC blood collection, primary tumor resected at CTC blood collection, liver metastasis, pre-cycle III CTC counts. ^b^ Multivariate COX regression model for OS with forward stepwise (Likelihood Ratio) including age, pre-cycle III CTC counts, baseline and pre-cycle III cfDNA, change of cfDNA1/2, change of CTC1/2, combined changes of CTC and cfDNA. Bold numbers indicate significant *p-*Value < 0.05.

## Supplementary Materials

The following are available online at https://www.mdpi.com/2072-6694/12/6/1352/s1, Figure S1: Timeline for serial analysis of palliative CT for advanced ESCC, Figure S2: Kaplan-Meier analysis of baseline CTC counts in (A) all ESCC cases, stratification (B) without previous treatment and (C) with previous treatment, Table S1: Statistics of baseline, pre-cycle III, post-cycle IV, post-CT, and relapse CTC counts and cfDNA level in ESCC, Table S2: ROC analysis of cfDNA and CTC and OS, Table S3: Definition of risk groups for combined changes of CTC and cfDNA, Table S4: Correlations of age, CTC2, cfDNA1, cfDNA2, change of CTC1/C2, change of cfDNA1/cfDNA2, and the combined change of CTC1/2 & cfDNA1/2, Table S5: Multivariable Cox analysis of change of baseline and pre-III CTC counts and cfDNA level with PFS, IR, and OS.
