# Supplementary material for: Liquid Biopsy Serial Monitoring of Treatment Responses and Relapse in Advanced Esophageal Squamous Cell Carcinoma
Source: Cancers (Basel). 2020 May 26;12(6):1352. doi: 10.3390/cancers12061352 (PMC7352685; doi:10.3390/cancers12061352)
Supplement: Supplementary file 1 [file cancers-12-01352-s001.pdf]

Figure S1: Timeline for serial analysis of palliative CT for advanced ESCC

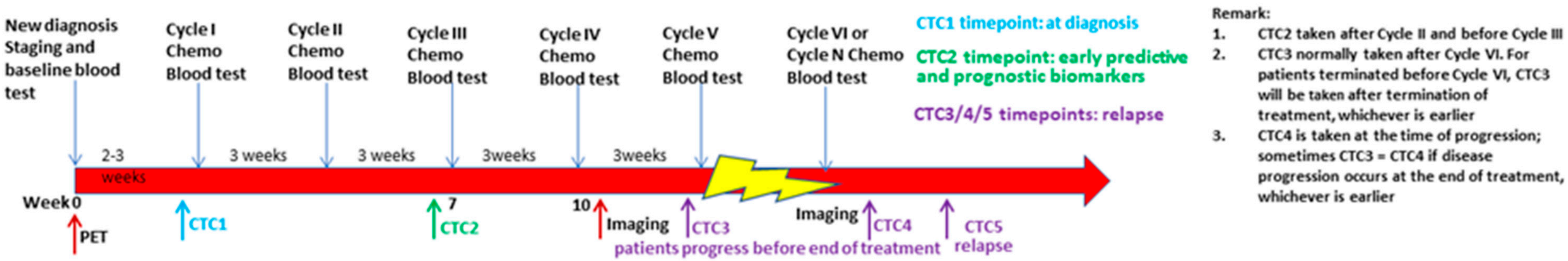

Figure S2: Kaplan-Meier analysis of baseline CTC counts with IR in (A) All ESCC cases, stratification by (B) without treatment and (C) with previous treatment

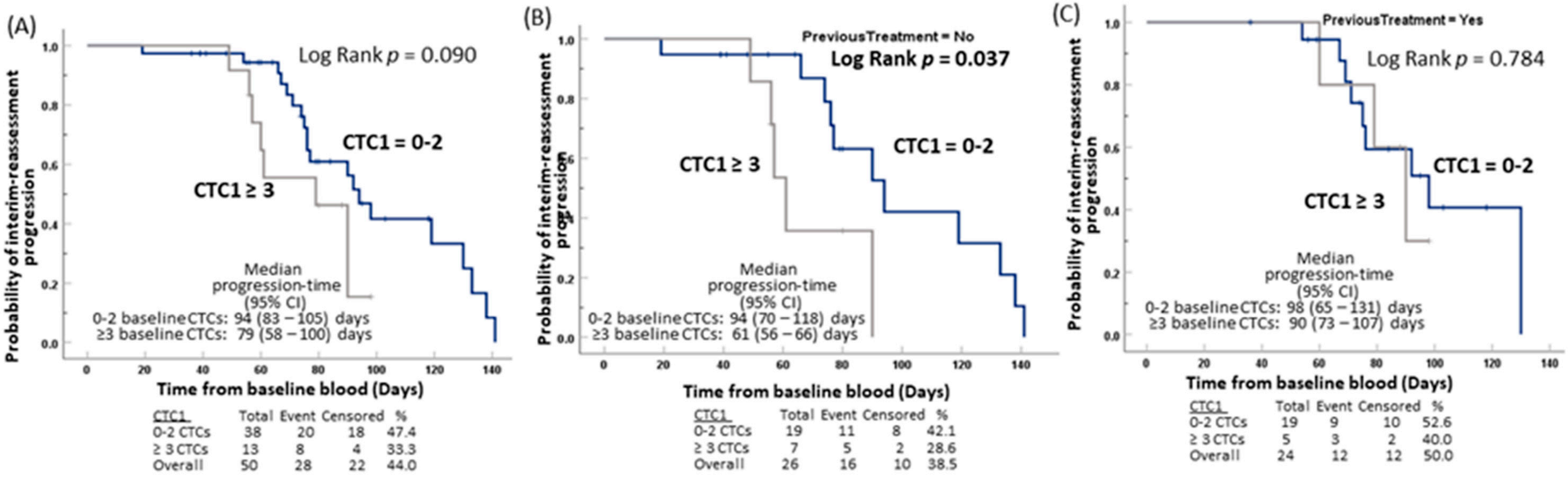

Table S1: Statistics of baseline, pre-cycle III, post-cycle IV, post-CT, and relapse CTC counts and cfDNA level in ESCC

Statistics

		Baseline CTC counts	Pre-III CTC counts	Post_IV CTC counts	Post-CT CTC counts	Relapse CTC counts	Baseline cfDNA hG copies/ml	Pre-III cfDNA hG copies/ml	Post-IV cfDNA hG copies/ml	Post-CT cfDNA hG copies/ml	Relapse cfDNA hG copies/ml
n	Evaluable	55	45	12	14	11	48	41	6	12	12
	Not applicable	2	12	45	43	46	9	16	51	45	45
Mean		2.31	2.47	0.92	0.79	3.45	4459.875	4190.754	2795.404	1694.293	16440.278
Median		1.00	1.00	1.00	0.00	1.00	3123.000	2175.758	3092.424	1578.636	3094.545
Std. Deviation		3.355	4.591	0.793	1.122	5.733	5347.5323	5367.9349	1084.7368	599.6916	30204.8151
Range		17	26	2	3	19	31169.0	22390.9	2486.7	2000.0	102536.7
Minimum		0	0	0	0	0	1067.0	315.2	1378.8	918.2	675.5
Maximum		17	26	2	3	19	32236.0	22706.1	3865.5	2918.2	103212.1
Percentiles	25	0.00	0.00	0.00	0.00	0.00	2022.250	1602.879	1508.333	1281.061	2432.955
	50	1.00	1.00	1.00	0.00	1.00	3123.000	2175.758	3092.424	1578.636	3094.545
	75	3.00	3.00	1.75	1.25	4.00	4712.500	3787.879	3810.227	2035.379	20093.182

Table S2: ROC analysis of cfDNA and CTC and OS

Parameters	AUROC	Threshold*	Sensitivity	Specificity
**Log cfDNA1 (*n*=48)**	0.627	3.360	0.667	0.533
**Log cfDNA2 (*n*=41)**	0.595	3.2817	0.714	0.462
**Log cfDNA4 (*n*=12)**	0.688	3.1936	0.75	0.625
**Log cfDNA5 (*n*=12)**	0.741	3.4384	0.778	0.667
**CTC2 (*n*=45)**	0.661	2.5	0.375	0.846
**CTC5 (*n*=11)**	0.732	0.5	0.857	0.75
**Integrated change of CTC1/2 & cfDNA1/2 (*n*=44)**	0.641	0.5	0.774	0.385

* Log_{10} transformed for cfDNA1 and cfDNA2 (copies of hG / ml of plasma) as a continuous variable. CTC counts for CTC1 and CTC2. Four risk groups (0-3) for integrated CTC1/2 & cfDNA1/2.

Table S3: Definition of risk groups for combined changes of CTC and cfDNA

Combinations	CTC1	CTC2	cfDNA1	cfDNA2	Total marks	Risk groups	No. of favorable changes (F) or unfavorable changes (U)
1	0	0	0	0	**0**	**0**	2F
2	1	0	0	0	**1**	**0**	2F
3	0	0	1	0	**1**	**0**	2F
4	1	0	1	0	**2**	**1**	**2F**
5	1	0	0	1	**2**	**1**	**1F + 1U**
6	0	1	0	0	**1**	**1**	**1F + 1U**
7	0	1	1	0	**2**	**1**	**1F + 1U**
8	0	0	0	1	**1**	**1**	**1F + 1U**
9	1	1	0	0	**2**	**1**	**1F + 1U**
10	0	0	1	1	**2**	**1**	**1F + 1U**
11	1	0	1	1	**3**	**1**	**1F + 1U**
12	1	1	1	0	**3**	**1**	**1F + 1U**
13	0	1	0	1	**2**	**2**	**2U**
14	0	1	1	1	**3**	**2**	**2U**
15	1	1	0	1	**3**	**2**	**2U**
16	1	1	1	1	**4**	**3**	**2U**

0 mark is given for CTC1/2 = 0-2; 1 mark is given for CTC1/2 >2.
0 mark is give for log cfDNA1<3.360; 1 mark is given for log cfDNA1 ≥ 3.360.
0 mark is give for log cfDNA1<3.2817; 1 mark is given for log cfDNA1 ≥ 3.2817.

Table S4: Correlations of age, CTC2, cfDNA1, cfDNA2, change of CTC1/2, change of cfDNA1/2, and the combined change of CTC1/2 & cfDNA1/2.

Correlations

Spearman's rho		Age	CTC2 (0 and 1 risk groups)	Baseline log cfDNA1	Pre-III log cfDNA2	Change of cfDNA1&2 (0 and 1 risk groups)	Change of CTC1/2 (0, 1, and 2 risk groups)	Change of combined CTC1/2 & cfDNA1/2
Age	Correlation Coefficient	1.000	-0.091	-0.146	-0.026	-0.102	-0.016	0.053
	Sig. (2-tailed)		0.553	0.321	0.873	0.530	0.919	0.733
	n	57	45	48	41	40	43	44
CTC2 (0 and 1 risk groups)	Correlation Coefficient	-0.091	1.000	0.222	0.209	0.172	.760**	.742**
	Sig. (2-tailed)	0.553		0.180	0.208	0.308	0.000	0.000
	n	45	45	38	38	37	43	41
Baseline log cfDNA1	Correlation Coefficient	-0.146	0.222	1.000	.485**	.648**	0.062	.331*
	Sig. (2-tailed)	0.321	0.180		0.002	0.000	0.718	0.035
	n	48	38	48	40	40	36	41
Pre-III log cfDNA2	Correlation Coefficient	-0.026	0.209	.485**	1.000	.729**	0.135	.633**
	Sig. (2-tailed)	0.873	0.208	0.002		0.000	0.432	0.000
	n	41	38	40	41	40	36	41
Change of cfDNA1&2 (0 and 1 risk groups)	Correlation Coefficient	-0.102	0.172	.648**	.729**	1.000	0.046	.508**
	Sig. (2-tailed)	0.530	0.308	0.000	0.000		0.791	0.001
	n	40	37	40	40	40	35	40
Change of CTC1/2 (0, 1, and 2 risk groups)	Correlation Coefficient	-0.016	.760**	0.062	0.135	0.046	1.000	.679**
	Sig. (2-tailed)	0.919	0.000	0.718	0.432	0.791		0.000
	n	43	43	36	36	35	43	39
Change of combined CTC1/2 & cfDNA1/2	Correlation Coefficient	0.053	.742**	.331*	.633**	.508**	.679**	1.000
	Sig. (2-tailed)	0.733	0.000	0.035	0.000	0.001	0.000	
	n	44	41	41	41	40	39	44

**. Correlation is significant at the 0.01 level (2-tailed).

*. Correlation is significant at the 0.05 level (2-tailed).

Table S5. Multivariable Cox analysis of change of baseline and pre-III CTC counts and cfDNA level with PFS, IR, and OS.

**Progression-Free Survival (PFS)**		
	**HR (95% CI)**	***P*-Value**
Age	1.032 (0.960 - 1.11)	0.392
Stage at CTC blood collection (IV vs III ref)	0.326 (0.091 – 1.169)	0.086
Change of CTC1/2		
Group 0	Reference	
Group 1	1.48 (0.549 – 3.989)	0.439
Group 2	31.2 (5.235 – 185.935)	1.58×10^{-4}
Change of cfDNA1/2		
Group 0	Reference	
Group 1	6.247 (2.051 – 19.026)	0.001
**Interim Reassessment (IR)**		
	HR (95% CI)	*P* value
Age	1.052 (0.949 – 1.166)	0.337
Stage at CTC blood collection (IV vs III ref)	0.434 (0.107 – 1.766)	0.029
Change of CTC1/2		
Group 0	Reference	
Group 1	1.036 (0.261 – 4.117)	0.96
Group 2	10.8 (1.581 – 73.794)	0.015
Change of cfDNA1/2		
Group 0	Reference	
Group 1	5.126 (1.184 – 22.197)	0.029
**Overall survival (OS)**		
	HR (95% CI)	*P* value
Age	0.987 (0.918 - 1.061)	0.713
Stage at CTC blood collection (IV vs III ref)	0.235 (0.064 – 0.864)	0.029
Change of CTC1/2		
Group 0	Reference	
Group 1	2.231 (0.752 – 6.618)	0.148
Group 2	9.945 (1.751 – 56.488)	0.01
Change of cfDNA1/2		
Group 0	Reference	
Group 1	24.494 (5.076 – 118.198)	6.8×10^{-5}
